# Notch1 signaling is involved in regulating Foxp3 expression in T-ALL

**DOI:** 10.1186/1475-2867-13-34

**Published:** 2013-04-11

**Authors:** Xiaodan Luo, Huo Tan, Yueqiao Zhou, Tiantian Xiao, Chunyan Wang, Yangqiu Li

**Affiliations:** 1 Department of Oncology & Hematology, the First Affiliated Hospital of Guangzhou Medical College, Guangzhou, 510230, China; 2 Institute of Hematology, Medical College, Jinan University, Guangzhou, China

**Keywords:** Notch1, T cell, Foxp3, Leukemia

## Abstract

**Background:**

T-cell acute lymphoblastic leukemia (T-ALL) is a highly aggressive hematologic malignancy. Immune tolerance induced by CD4^+^CD25^+^ regulatory T cells (Tregs) with high expression of Foxp3 is an important hypothesis for poor therapy response. Notch1 signaling is thought to be involved in the pathogenesis of this disease. Crosstalk between Notch and Foxp3^+^Tregs induced immune tolerance is unknown in T-ALL. We studied Foxp3 and Notch1 expression in vivo and in vitro, and analyzed the biological characteristics of T-ALL cell line systematically after Notch inhibition and explored the crosstalk between Notch signaling and Foxp3 expression.

**Methods:**

In vivo, we established T-ALL murine model by Jurkat cells transplantation to severe combined immunodeficiency (SCID) mice. Notch1 and Foxp3 expression was detected. In vitro, we used γ-secretase inhibitor N-S-phenyl-glycine-t-butyl ester (DAPT) to block Notch1 signaling in Jurkat cells. Notch1, Hes-1 and Foxp3 genes and protein expression were detected by PCR and western blotting, respectively. The proliferation pattern, cell cycle and viability of Jurkat cells after DAPT treatment were studied. Protein expression of Notch1 target genes including *NF-κB, p-ERK1/2* and *STAT1* were determined.

**Results:**

We show that engraftment of Jurkat cells in SCID mice occurred in 8 of 10 samples (80%), producing disseminated human neoplastic lymphocytes in PB, bone marrow or infiltrated organs. Notch1 and Foxp3 expression were higher in T-ALL mice than normal mice. In vitro, Jurkat cells expressed Notch1 and more Foxp3 than normal peripheral blood mononuclear cells (PBMCs) in both mRNA and protein levels. Blocking Notch1 signal by DAPT inhibited the proliferation of Jurkat cells and induced G0/G1 phase cell cycle arrest and apoptosis. Foxp3 as well as p-ERK1/2, STAT1 and NF-κB expression was down regulated after DAPT treatment.

**Conclusions:**

These findings indicate that regulation of Foxp3 expression does involve Notch signaling, and they may cooperatively regulate T cell proliferation in T-ALL.

## Background

T-cell acute lymphoblastic leukemia (T-ALL) is a highly aggressive hematologic malignancy that represents 10% to 15% of pediatric and 25% of adult acute lymphoblastic leukemia cases [1-6]. Compared to B-cell acute lymphoblastic leukemia (B-ALL), patients with T-ALL commonly present large tumor burdens at diagnosis and invariably poor outcomes even after intensified chemotherapy. The specific biological and molecular mechanisms that account for the aggressiveness and poor therapy response in T-ALL remain unclear and T-ALL cells induced immune tolerance is an important hypothesis [1-6]. Some reports showed that T-ALL cells are derived from regulatory T cells (Tregs), which suppress the reaction of lymphocytes to tumor antigens and induce immune tolerance and malignant neoplasm progression. Foxp3 is a specific and important marker for Tregs. Recent reports showed that the aggressiveness and poor outcome of T-ALL are closely related to the large number of Tregs and high expressions of Foxp3 in tumor microenvironment [4,7,8].

Notch1 is more and more concerned in T-ALL and activating mutations in the Notch1 gene are present in over 50% of human T-ALL cases making Notch1 the most prominent oncogene specifically involved in the pathogenesis of this disease [2,3,9-12]. The Notch pathway regulates T cell proliferation and development and therefore is critical for ensuring the proper differentiation of T cell [3,10,12-15]. “Gain of function” mutation within Notch1 was found in both T-ALL patient samples and murine T-ALL models [3,10,12,16]. Activation of Notch receptors is triggered by interaction with Notch ligands Jagged and Delta-like on adjacent cells, which results in proteolytic cleavage of Notch and subsequent release of the intracellular domain (IC) [2,3,9-12]. Notch-IC is then transported into the nucleus and associates with *RBP-Jk/CBF-1*, resulting in the activation of target genes including the *Hes* family of proteins [17-19]. Inhibition of Notch1 signaling using γ-secretase inhibitors (GSIs) induced rapid clearance of Notch-IC and transcriptional down regulation of Notch1 target genes. The precise mechanism by which Notch activation leads to T-ALL is still unclear. Key pathways include the PI3-kinase/Akt, mTOR and NF-κB. Zou J et al. report that Notch1 is required for hypoxia-induced proliferation, invasion and chemoresistance of T-cell acute lymphoblastic leukemia cells [1,3,14,15,20-23]. Crosstalk between Notch and these pathways is also incompletely understood and probably occurs at several levels.

Several studies have implicated the participation of Notch signaling in Treg differentiation and suppressor function. Overexpression of Notch ligand can induce Treg and Foxp3^+^Tregs express high levels of Notch1 [8,24-27]. Ou-yang showed that Notch1 signaling can activate the *Foxp3* promoter and *Hes1* might be an important regulatory factor at the transcriptional level in the lineage determination of Tregs development [8]. However, very few reports have shown the association between Notch1 and Foxp3 and the crosstalk between them is unknown. In this study, we show not only Notch1 and Foxp3 expression in T-ALL group both in vivo and in vitro, but also the biological characteristics of T-ALL cell line as Notch1 and Foxp3 expression was inhibited. Blocking Notch1 signaling by GSI N-S-phenyl-glycine-t-butyl ester (DAPT) inhibited the expressions of Notch1 and Foxp3 in Jurkat cell line, inducing apoptosis of Jurkat cells. Protein levels of NF-κB, p-ERK1/2 and STAT1 were also decreased in Notch1 inhibited Jurkat cells. These findings suggested that inhibition of Foxp3 expression does involve Notch signaling, and it may be mediated by the regulation of NF-κB, p-ERK1/2 and STAT1 pathways.

## Results

### *Engraftment in* Non-obese diabetic (NOD)/Severe combined immunodeficiency (SCID) *mice*

**Figure 1:**
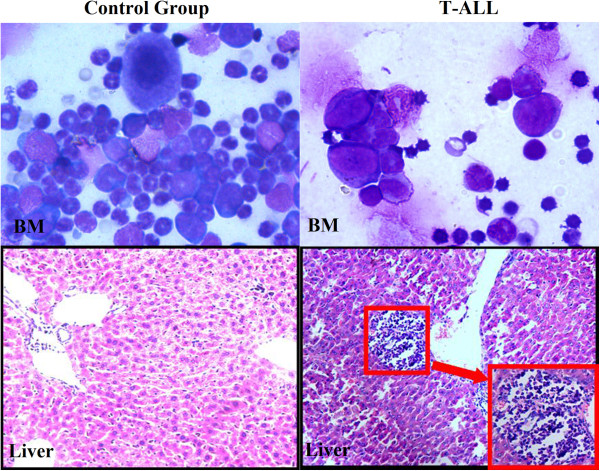
**Morphology of cells of bone marrow smear and liver.** Morphology of cells of bone marrow was studied under microscopy (Wright-Giemsa, ×1000) and liver was inspected for signs of leukemic infiltration (HE, ×400). Neoplastic cells were found in T-ALL group.

Engraftment occurred in 8 of 10 samples (80%), producing disseminated human neoplastic lymphocytes in peripheral blood (PB), and bone marrow or infiltrated organs. The median mouse survival duration was 57.3 days (range, 40 to 60). In most cases, a gradual increase in circulating neoplastic cells was seen; in some cases, no neoplastic cells were detected in peripheral blood and evidence of engraftment was obtained at necropsy. Jurkat cell like neoplastic cells were found in PB, bone marrow smear (Figure 1 top panel showed one representative bone marrow smear, the neoplastic cells in the T-ALL) and infiltrated in other organs including liver, spleen, lung, kidney and gastro intestine. These results were confirmed with hematoxylin and eosin (H&E) staining of mouse liver (representative data shown in Figure 1 low panel).

#### Notch1 and Foxp3 gene and protein expression were higher in T-ALL mice than normal mice

**Figure 2:**
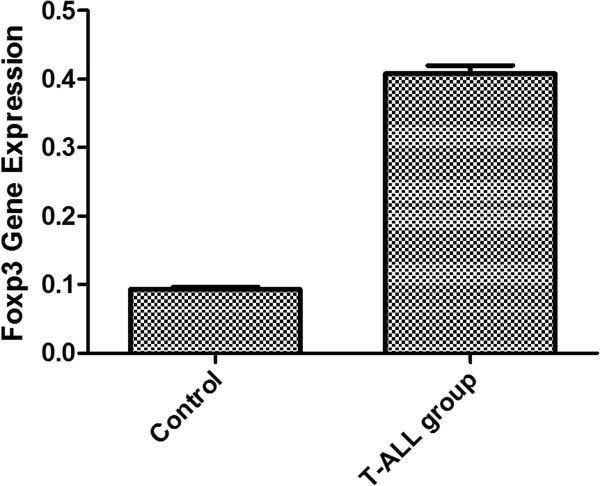
***Foxp3 *gene expression.** We assessed *Notch1* and *Foxp3* expression in PB in T-ALL mice and the control by RT-PCR. Both *Notch1* and *Foxp3* were detected in T-ALL group, while in the control group, *Notch1* was not detected. The expression of *Foxp3* in T-ALL group was significantly higher than the control group (*P* < 0.05).

**Figure 3:**
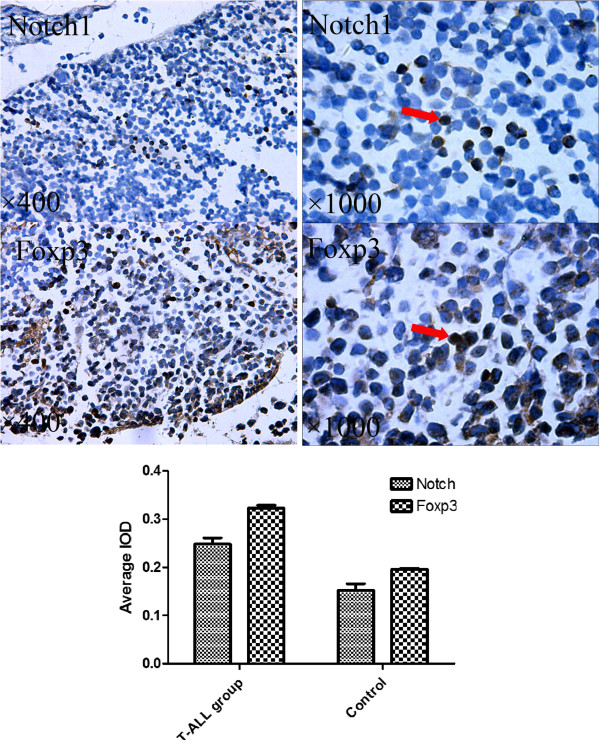
**Notch1 and Foxp3 protein expression in tumor tissues.** Tumor tissues in T-ALL mice were collected immunohistochemical assay. Samples were treated with rabbit polyclonal anti-Notch1 and anti-Foxp3. Both Notch1 and Foxp3 protein were detected in T-ALL mice and Foxp3 protein was detected mostly around tumor tissues. Image-pro plus was used to evaluate the expressions of Notch1 and Foxp3 through immunohistochemical staining. Protein expression was measured in IOD. Notch1 and Foxp3 protein expression in T-ALL mice were significantly higher than the control (*P* < 0.05).

We assessed *Notch1* and *Foxp3* expression in PB in T-ALL mice and the control by RT-PCR. Both *Notch1* and *Foxp3* were detected in T-ALL group, while in the control group, *Notch1* was not detected and the expression of *Foxp3* was significantly lower than T-ALL group (*P* < 0.05) (Figure 2). We next assessed Notch1 and Foxp3 protein expression in different organs. Both Notch1 and Foxp3 protein were detected in organs in normal mice and T-ALL mice. Foxp3 protein was detected mostly around tumor tissues (Figure 3). Notch1 and Foxp3 protein expression in T-ALL mice were significantly higher than the control (*P* < 0.05).

#### Jurkat cells express Notch1 and more Foxp3 than normal PBMCs

**Figure 4:**
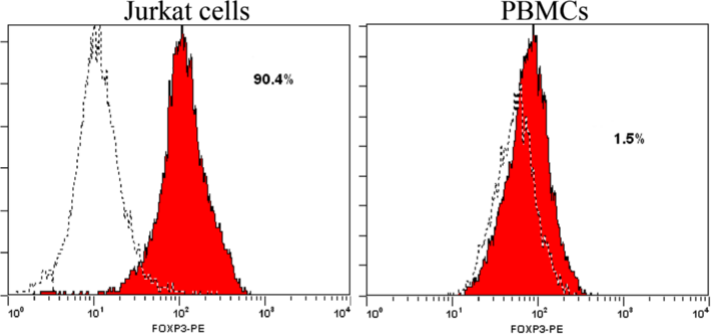
**Foxp3 expression in Jurkat cells and PBMCs.** The expression of Foxp3 in Jurkat cells and PBMCs was analyzed by flow cytometry. Foxp3-expressing jurkat cells were much more than Foxp3-expressing PBMCs (*P* < 0.05).

We assessed the expression of Notch1 in Jurkat cells and PBMCs from healthy donors by RT-PCR and western-blot. Jurkat cells expressed Notch1. The expression of Notch1-Cleaved protein was 48.03 ± 1.57% by western-blot. We also assessed the expression of Foxp3 in Jurkat cells and PBMCs by Real-time PCR and flow cytometry. As shown in Figure 4, Foxp3-expressing jurkat cells were 88 ± 2.5%, which is much more than Foxp3-expressing PBMCs (5 ± 3.5%) (*P* < 0.05).

#### Blocking Notch1 signal by DAPT inhibits the proliferation of Jurkat cells

To study the characteristics of Jurkat cells after DAPT treatment for 48 hours, cells were viewed under microscope. Jurkat cells without DAPT were usually round with clear areas of cytoplasm and nuclear and proliferated into cell clusters. However, Jurkat cells with DAPT were shown difficult to proliferate into cell clusters.

**Figure 5:**
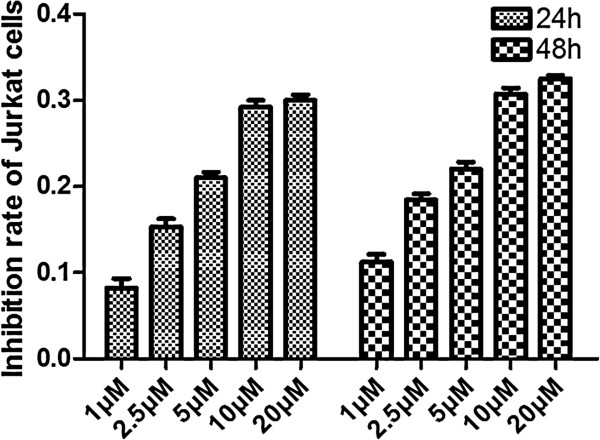
**Jurkat cell viability after DAPT treatment.** Jurkat cell viability assay was performed by CCK8 method and the inhibition rate was analyzed. Jurkat cells were treated with increasing concentrations of DAPT (1, 2.5, 5, 10, 20 μM). The proliferation rate of Jurkat cell increased as the concentration of DAPT increased after 24 and 48 hours stimulation, especially the 48-hour time point. The inhibition was in a concentration-dependent manner with the greatest effect observed at 20 μM DAPT.

We next proved that DAPT could inhibit Jurkat cell proliferation by CCK8 method. Jurkat cells were treated with increasing concentrations of DAPT (1, 2.5, 5, 10, 20 μM) for 4, 8, 12, 24, 48 and 72 hours, respectively. After stimulated for 4, 8 and 12 hours, Jurkat cells proliferated as those treated with DMSO alone. Jurkat cell proliferation was inhibited more and more remarkably as the concentration of DAPT increased after they were stimulated for 24 and 48 hours compared to DMSO control. However, after 72 hours stimulation, the proliferation of Jurkat cells was not inhibited by DAPT. These results indicated that DAPT could inhibit Jurkat cell proliferation only after 24 and 48 hours stimulation, especially the 48-hour time point and the inhibition was in a concentration-dependent manner with the greatest effect observed at a concentration of 20 μM, and the inhibition rate was as high as 33 ± 2.3% (Figure 5).

**Figure 6:**
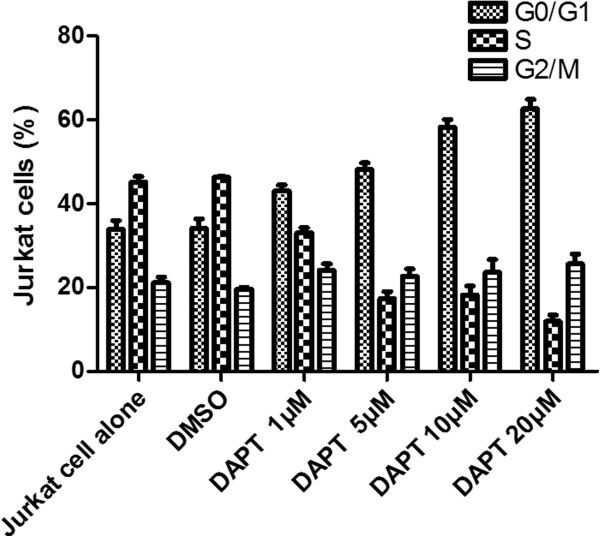
**Jurkat cells cycle after DAPT treatment.** The effect of DAPT on cell cycle was determined by flow cytometric analysis. Jurkat cells were treated with increasing concentrations of DAPT (1, 5, 10, 20 μM) for 48 hours. The percentage of Jurkat cells in the sub-G0/G1 phase increased significantly while in S and G2/M phase decreased compared to the cells alone and DMSO control (*P* < 0.05).

To study the effect of DAPT on cell cycle, we further stimulated Jurkat cells with increasing concentrations of DAPT (1, 5, 10, 20 μM) for 48 hours. The results showed that the percentage of Jurkat cells in the sub-G0/G1 phase increased significantly while in S and G2/M phase decreased (*P* < 0.05). Increasing concentrations of DAPT induced G0/G1 phase cell cycle arrest in more Jurkat cells, indicative of apoptosis (Figure 6).

#### Blocking Notch1 signal by DAPT induces Jurkat cells apoptosis

**Figure 7:**
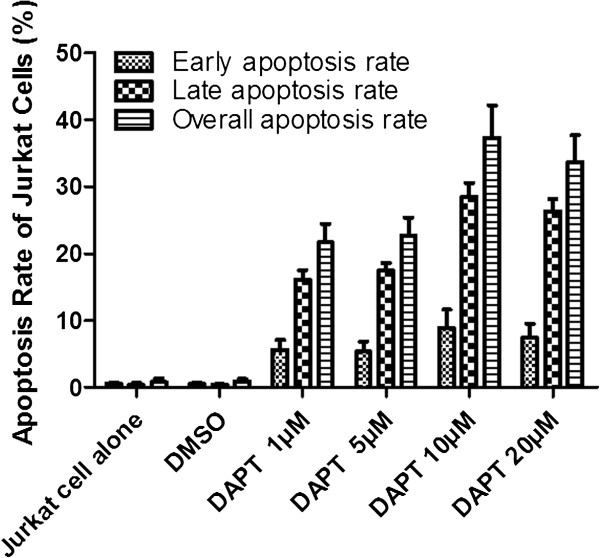
**Apoptosis rate of Jurkat cells after DAPT treatment.** The effects of DAPT on apoptosis were analyzed by annexin V/PI staining. Jurkat cells were treated with increasing concentrations of DAPT (1, 5, 10, 20 μM) and the results showed that the early and late apoptosis rate increased as DAPT concentrations increased compared to the cells alone and DMSO control (*P* < 0.05).

To further document the effects of DAPT on apoptosis, analysis by annexin V/PI staining was performed after treatment with increasing concentrations of DAPT (1, 5, 10, 20 μM). The results showed an increase in apoptotic cells in Jurkat cells as the concentration of DAPT increased. The apoptosis rate with DAPT (1, 5, 10, 20 μM) was 21.7 ± 2.77%, 22.7 ± 2.71%, 37.3 ± 4.9% and 33.7 ± 4%, respectively, compared with 0.84 ± 0.38% for control (Figure 7) (*P* < 0.05).

#### Notch1 and Hes-1 gene and protein expression is down regulated

Jurkat cells were treated with increasing concentrations of DAPT (1, 5, 10, 20 μM) for 48 hours and RT-PCR was used to assess *Notch1* gene expression. *Notch1* was down regulated in Jurkat cells with DAPT treatment compared with cells with DMSO.

**Figure 8:**
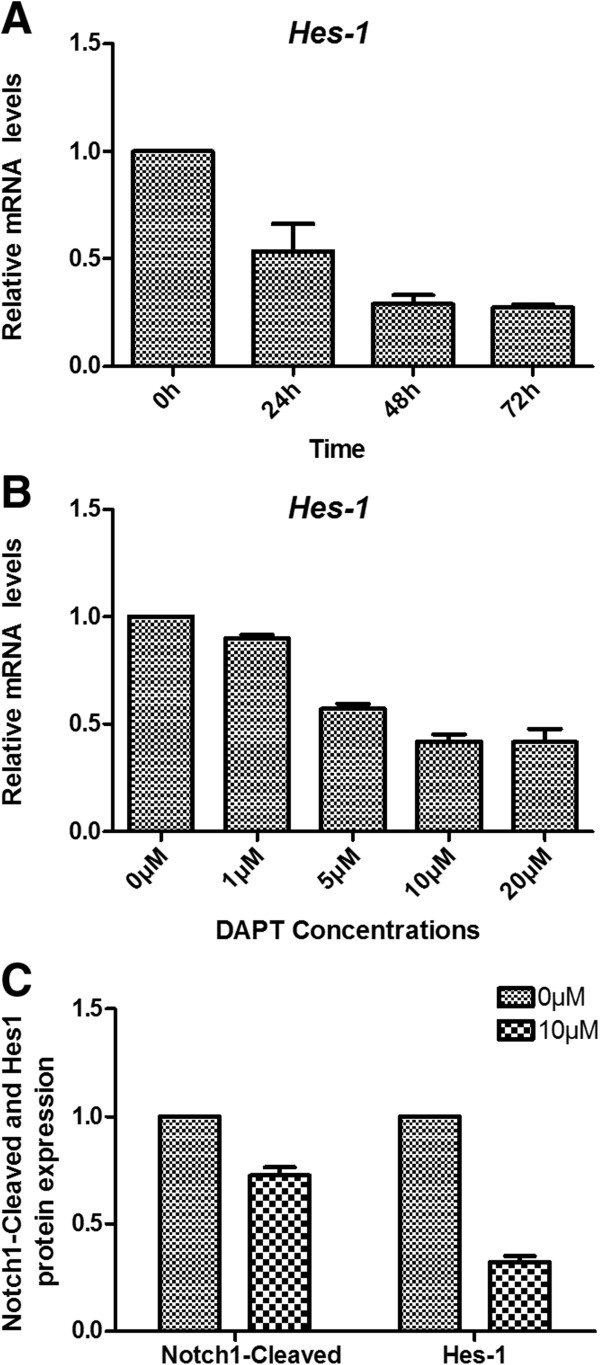
**Expression of *Hes1*, Notch1-Cleaved and Hes-1 protein after DAPT treatment. A**: Real-Time PCR was used to assess *Hes-1* gene expression. *Hes1* was down regulated in Jurkat cells treated with 10 μM DAPT for 24, 48 and 72 hours, especially for 48 hours. **B**: At 48 hours time point, DAPT had the greatest effect on *Hes1* expression when its concentrations were 10 μM. **C**: Notch1-Cleaved and Hes-1 protein expression was assessed by western blot. At 48-hours treatment with 10 μM DAPT, Notch1-Cleaved and Hes-1 protein expression was lower than the control group (*P* < 0.05).

*Hes1* is one of the target genes of Notch1 signal. Real-Time PCR was used to assess *Hes-1* expression. *Hes1* was down regulated in Jurkat cells treated with 10 μM DAPT for 24, 48 and 72 hours and gene expression was 53.59 ± 12.7%, 28.95 ± 4.2% and 27.35 ± 1.4%, respectively, compared to the control (*P* < 0.05). *Hes1* expression had a significant decrease after 48 hours treatment with DAPT (Figure 8A). At this time point, *Hes1* expression was 90.12 ± 1.4%, 57.3 ± 2.2%, 42.1 ± 3.3% and 41.8 ± 6%, respectively, in Jurkat cells with different concentrations of DAPT (1, 5, 10, 20 μM) compared to the control (*P* < 0.05). DAPT had the greatest effect on *Hes1* expression when its concentrations were 10 μM (Figure 8B). We next sought to assess the Notch1-Cleaved and Hes-1 protein by western blot. At 48 hours treatment with 10 μM DAPT, Notch1-Cleaved and Hes-1 protein expression was 72.5 ± 3.8% and 32.1 ± 2.9% (*P* < 0.05), respectively, which was lower than the control group. Therefore, DAPT can inhibit Notch1-Cleaved and Hes-1 protein expression (Figure 8C).

#### In vitro DAPT treatment block Foxp3 expression

**Figure 9:**
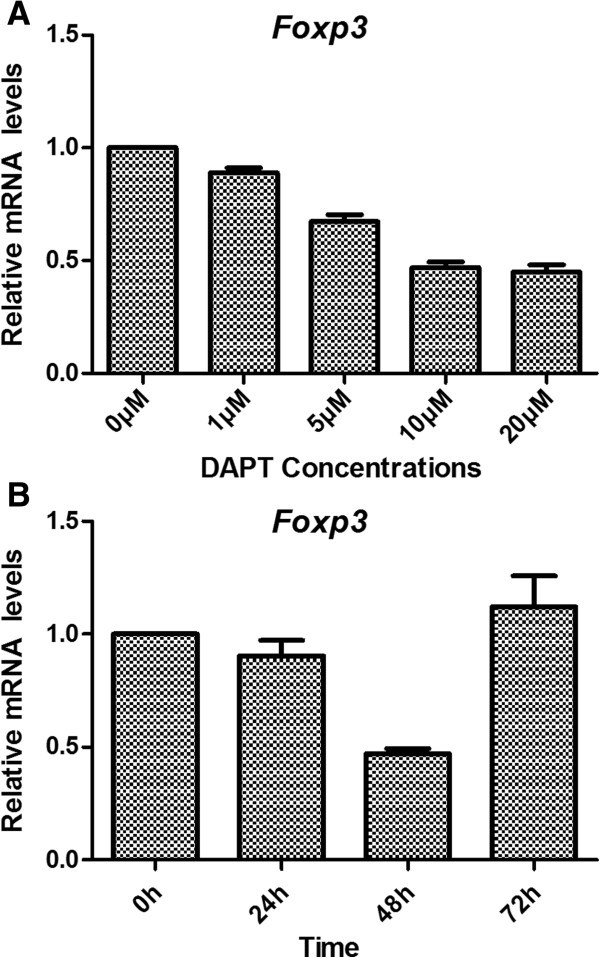
***Foxp3 *gene expression after DAPT treatment. A**: *Foxp3* gene expression was down regulated as the concentrations of DAPT increased compared to the control. DAPT had the greatest effect on *Foxp3* expression when DAPT was 10 μM. **B**: Jurkat cells were treated with DPAT at 10 μM for 24, 48 and 72 hours and DAPT had the greatest effect at 48-hours time point. After 72 hours, *Foxp3* expression was up regulated.

As reported by Ouyang, Notch1 signaling can activate the *Foxp3* promoter. We then assessed Foxp3 gene and protein expression after Notch1 inhibition. *Foxp3* expression was 89 ± 2.1%, 67.3 ± 3%, 46.98 ± 2.5% and 45 ± 3.2% when DAPT was at 1, 5, 10 and 20 μM, respectively. *Foxp3* expression was down regulated as the concentrations of DAPT increased compared to the control (*P* < 0.05) (Figure 9A). *Foxp3* expression was 90.5 ± 6.7%, 46.98 ± 2.5% and 112 ± 14% (*P* < 0.05) when Jurkat cells were treated with DPAT at 10 μM for 24, 48 and 72 hours, respectively. This showed that DAPT had the greatest effect on *Foxp3* expression when Jurkat cells were treated with DAPT at 10 μM for 48 hours. In contrast, after 72 hours, *Foxp3* expression was up regulated (Figure 9B). Flow cytometry was used to assess the Foxp3 protein expression and the result showed that DAPT could also inhibit Foxp3 protein expression. Foxp3 protein expression was 65.5 ± 3.5%, 60.9 ± 2.4%, 58.8 ± 2.8% and 50.7 ± 1.9% when Jurkat cells were treated with DAPT (1, 5, 10, 20 μM) for 48 hours. Similar to the gene expression, Foxp3 protein expression began to increase at 72 hours treatment with 10 μM and 20 μM DAPT. This inhibition effect was similar to what was observed in Jurkat cells, which began to proliferate after 72 hours treatment with DAPT.

#### The expression of NF-κB, p-ERK1/2 and STAT1 are deregulated in Jurkat cells after Notch1 signal inhibition

**Figure 10:**
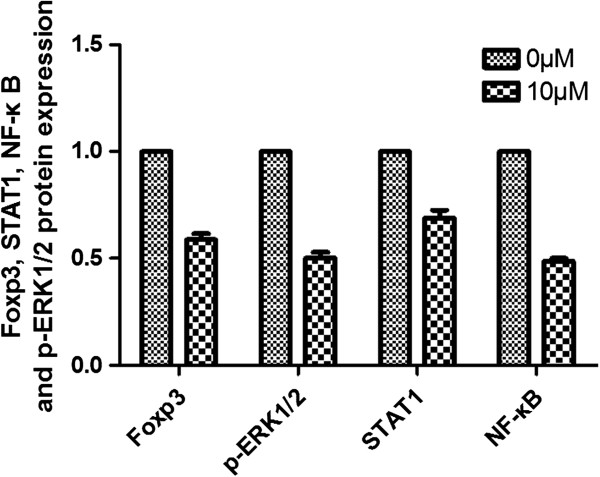
**p-ERK1/2, STAT1 and NF-κB protein expression.** p-ERK1/2, STAT1 and NF-κB protein expression assessed by western blot. Similar to Foxp3 protein expression, p-ERK1/2, STAT1 and NF-κB protein expression was down regulated when Jurkat cells were treated with 10 μM DAPT for 48 hours.

*p-ERK1/2, STAT1* and *NF-κB* are Notch1 target genes. To determine whether Notch1 inhibition was related to the expression of p-ERK1/2, STAT1 and NF-κB, we assessed the protein expression after Notch1 inhibition by DAPT. Similar to what was observed in Notch1, Hes-1 and Foxp3 expression, p-ERK1/2, STAT1 and NF-κB protein expression was down regulated when Jurkat cells were treated with 10 μM DAPT for 48 hours. p-ERK1/2, STAT1 and NF-κB protein expression was 50.1 ± 2.9%, 68.8 ± 3.8% and 48.7 ± 1.4%, respectively (*P* < 0.05) (Figure 10).

## Discussion

Foxp3^+^Tregs play an important role in regulating the immune system by suppressing self-reactive T cells that have escaped negative selection in the thymus as well as hyperactive T cells that are induced during excessive immune responses in peripheral lymphoid tissues. On one hand, mutation or deletion of the gene encoding Foxp3 causes severe autoimmune diseases in both human and mice, due to a malfunction of Tregs. On the other hand, ectopic expression of Foxp3 in conventional T-cells confers immunosuppressive activities, suppressing normal T cell immunity against tumor [7,8,14,28]. Foxp3 was also expressed in some T-ALL cells and was a specific marker of T-ALL. Bonzheim et al. [29] found that T cells within the T-ALL cell infiltrate were mainly Foxp3 expressing cells, and only a few tumor infiltrating reactive lymphocytes could be observed. In the study of Karube et al. [7], Foxp3 expression was confirmed in T-ALL and Foxp3^+^ T-ALL cells might suppress tumor immunity and promote tumor growth. Roncador et al. [30] also reported that Foxp3^+^ T-ALL showed a more aggressive clinical course than Foxp3^-^ T-ALL. In our study, we established T-ALL murine model with SCID mice and found that Foxp3 expression increased in T-ALL mice compared to normal mice. We then detected Foxp3 expression in both human T cell leukemia cell line and PBMCs from healthy donors. We found that Foxp3 expression was higher in Jurkat cells than in PBMCs. The results from in vivo and in vitro indicated that Foxp3 expression was associated with T-ALL, which was compatible with what was found in Karube’s study.

Recently, deregulation of Notch signaling has been linked to the development of T-ALL. The recent identification of activating mutations in Notch1 in the majority of T-ALL has brought major interest towards targeting the Notch signaling pathway in this disease [3,12,15,16,22,31,32]. The fundamental importance of Notch1 mutations in T-ALL is highlighted by the potential role of Notch1 as a molecular therapeutic target for the treatment of this disease. Pharmacologic inhibition effectively abrogates oncogenic Notch1 signaling in T-ALL cells. GSIs induced rapid clearance of intracellular activated Notch1 protein and transcriptional downregulation of Notch1 target genes [1,3,4,11]. In our study, the biological characteristics of Jurkat cells as well as Notch1 target gene expression were studied after pharmacologic inhibition of Notch signaling using GSI. DAPT inhibited the proliferation of Jurkat cells. As DAPT concentrations increased, the viability of Jurkat cells decreased. DAPT induced G0/G1 phase cell cycle arrest in Jurkat cells, which resulted in apoptosis. We further detected Notch1 and Hes-1 gene and protein expression after DAPT treatment. *Notch1* and *Hes-1* were down regulated and Notch1-Cleaved and Hes-1 protein expression significantly decreased compared to the control group. These suggested that DAPT could inhibit Notch1 signaling by down regulating Notch1 target genes and induce Jurkat cell apoptosis.

Except for the aberrant Notch mutation that induces T-ALL, immunosuppression in T-ALL has also been the subject of many discussions. Karube et al. [7] indicated that T-ALL cells might function as Treg-like cells and induce the immunosuppressive state especially in Foxp3^+^ cases. However, the mechanisms leading to immune tolerance by Foxp3^+^ Tregs in T-ALL remain largely unknown. Recently, Notch and its ligands have been implicated in the regulation and differentiation of various CD4^+^T-helper cells [2,3,9-11,33]. Is Notch1 also involved in regulating Foxp3? Samon et al. [34] provided evidences that Foxp3 was a downstream target of Notch signaling. Pharmacologic inhibition of Notch signaling using GSIs blocked the up-regulation of Foxp3 target genes and induces Foxp3 expression [34]. GSIs also inhibited the binding of Notch1, CSL, and Smad to conserved binding sites in the Foxp3 promoter. Moreover, in vivo GSIs treatment down-regulated Foxp3 expression and resulted in a spontaneous lymphocyte infiltration of the liver in mice. Ou-Yang et al. [8] showed that Notch signaling could modulate the Foxp3 promoter through *RBP-J-* and *Hes1-*dependent mechanisms and Notch signaling might be involved in the development and function of Tregs through regulating Foxp3 expression. In order to study the association between Notch1 and Foxp3, we detected Foxp3 gene and protein expression in Jurkat cells treated with DAPT. *Notch1* and *Hes-1* had a significant drop and *Foxp3* was down regulated at the same time point. This suggested that Notch1 signaling was involved in regulating Foxp3 expression in Jurkat cell.

These previous findings led us to explore the crosstalk between Notch1 and Foxp3 in Jurkat cells. We hypothesized that activated Notch1 might increase Foxp3 expression by up regulating some target genes. Previous reports [25-27,35,36] have suggested that Notch can display both stimulatory and inhibitory control of NF-κB activity. It has been hypothesized that activated Notch in T cells may result in constitutive NF-κB activation, leading to T-cell leukemia/lymphoma. *NF-κB* as well as *p-ERK1/2 and STAT1* are Notch1 target genes. We assessed the protein expression of NF-κB, p-ERK1/2 and STAT1. The result showed that the protein expression was down regulated after Notch1 was inhibited by DAPT. These suggested that inhibition of Foxp3 expression involved Notch signaling, and it may be mediated by regulation of NF-κB, p-ERK1/2 and STAT1 pathways.

## Conclusions

In summary, this study systematically showed Notch1 and Foxp3 expression as well as its impact on T-ALL cell proliferation and development. By studying the biological change of Jurkat cells after Notch1 inhibition, we showed that down regulation of Notch1 and Foxp3 could induce Jurkat cell apoptosis. The association between Notch1 and Foxp3 was another important subject of this study. Notch signaling is involved in regulating Foxp3 expression in Jurkat cells and it could be mediated by regulation of NF-κB, p-ERK1/2 and STAT1 pathways. These results together indicated that Notch1 signaling that induces Foxp3 expression might be associated with immunosuppression state in T-ALL.

## Materials and methods

### Ethics statement

Peripheral blood samples in this study were collected from healthy donors in hospital. All participants are residence in our country. Samples were collected for diagnostic purposes. After the original purpose has been achieved, the residual samples were used for research only without additional charges. All participants were informed of full information about the purposes of the sampling, and/or the plan of the research proposal. All participants have signed the informed consent before enrolling in this study. The informed consent is not only for this study, but also for other studies in which human blood samples are needed. All signed consent is in Chinese and documented. This ethics approval was obtained from Committee on the Ethics of the First Affiliated Hospital of Guangzhou Medical College (Permit number: 2012–41).

This study (Establishment of T-cell acute lymphoblastic leukemia murine model using NOD/SCID mice and the assessment of Notch1 and Foxp3 expression in this model) was carried out in strict accordance with the recommendations in the Guide for the Care and Use of Laboratory Animals of Guangzhou Medical College. Animals were purchased from Animal experimental center, Guangdong, China. The protocol was approved by the Committee on the Ethics of the First Affiliated Hospital of Guangzhou Medical College (Permit number: 2012–41). Mice that developed T-ALL may have experienced discomfort. Signs included increased abdominal girth from tumor infiltration, dehydration, decreased activity and cachexia. Mice with T-ALL were susceptible to infection. Mice were observed daily by laboratory staff and animal technicians and weighed once a week to detect weight loss. If the mice decompensated, they were immediately euthanized by CO_2_ to minimize suffering.

### Cell line and samples

Jurkat cells are a human T cell leukemia cell line that constitutively expresses IC [37] and, therefore, were used in this study. Jurkat cells were purchased from American Type Cell Culture (ATCC) and maintained according to the ATCC protocol. As described elsewhere [38], peripheral blood mononuclear cells (PBMCs) were separated from fresh blood samples by density gradient centrifugation. Red blood cells were removed from splenocytes using ammonium chloride lysis buffer.

### Experimental animal and procedures

NOD/ SCID mice (Animal experimental center, Guangdong, China) were used. Twenty female mice aged 5 weeks were maintained in a specific pathogen-free environment. Twenty mice weight 10.45 g to 11.62 g (median weight 11.12 g) were divided into T-ALL group and the control group randomly with 10 mice in each group. Physical randomisation procedure using random number tables was performed to assign mice to each group. Mice were injected intraperitoneally with cyclophosphamide (100 mg/kg/d) [39] for 2 days. In T-ALL group, Jurkat cells in the logarithmic phase of growth were then collected and transferred intravenously (5× 10^6^/mouse/day) through tail vein for 2 days. Mice in the control group were injected with physiological saline. Engraftment of Jurkat cells in mice was monitored by serial tail vein sampling every 7 days. This was done without anesthesia. To warm the tail with the aid of a heat lamp to increase obtainable blood volume before tail nicking. Decompensated mice were euthanized by CO_2_, when PB infiltration or clinical status like suggested engraftment. Mice were exposed to a CO_2_ concentration of 70% and maintained for 2 minutes after apparent clinical death. Other mice were evaluated for 60 days before sacrifice and necropsy. PB was collected for *Notch1* and *Foxp3* gene expression. Internal organs were inspected for signs of leukemic infiltration. Tissues from infiltrated organs were collected for Notch1 and Foxp3 protein expression. Single-cell suspensions from bone marrow were also prepared for flow cytometric analysis.

### Histopathology and immunochemistry

Samples of tissues were immersed in 10% neutral formalin. Formalin-preserved specimens were then embedded in paraffin, cut into 5 μm sections, and stained with H&E for histopathology examination. For immunohistochemical assay, paraffin-embedded sections were dewaxed, rehydrated and incubated with 0.5% hydrogen peroxide in methanol to quench endogenous tissue peroxidase. Sections were incubated with pepsin for 45 min for antigen retrieval. After blocking nonspecific sites with 1% BSA in PBS, sections were treated with rabbit polyclonal anti-Notch1 and anti-Foxp3 (Abcam) overnight and then with appropriate biotin-conjugated secondary antibodies for 20 min. Image-pro plus was used to evaluate the expressions of Notch1 and Foxp3 using immunohistochemical staining. Protein expression was measured in integrated optical density (IOD).

### Reverse-transcription PCR (RT-PCR) and real-time PCR

Total RNA was isolated from 1-5 × 10^6^ Jurkat cells using the RNeasy kit (Qiagen) and was resuspended in 40 μl RNase free H_2_O. First-strand cDNA synthesis was performed with oligo (dT) as primer. Notch1-IC primers were 5^′^-TTCCCTGAGGGCTTCAAAGT-3^′^ (forward) and 5^′^-CCCGCTACTCACGCTCTG-3^′^ (reverse). The primers of extracellular domain of Notch1 were 5^′^-CCGGTGAGACCTGCCTGAAT-3^′^ (forward) and 5^′^-GCACTTGTACTCCGTCAGCG-3^′^ (reverse). RT-PCR for Notch1 was performed in duplicate (30 cycles of 98°C for 10 s, 55°C for 30 s, and 72°C for 45 s). PCR products were subjected to 2% agarose gel electrophoresis and relative gene expression was measured in grey value. Foxp3 primers were 5^′^-ACTGACCAAGGCTTCATCTGTG-3^′^ (forward) and 5^′^-GGAACTCTGGGAATGTGCTGT-3^′^ (reverse). RT-PCR for Foxp3 mRNA expression (in vivo experiment) was performed as before. Real-time PCR for Foxp3 mRNA quantification was performed in duplicate with the Sofast EvaGreen Supermix (Bio-Rad) (40 cycles of 95°C for 30 s, 95°C for 5 s, and 56°C for 10 s, 65-95°C for 10 s). *Hes-1* primers were 5^′^-GGCTAAGGTGTTTGGAGGCT-3^′^ (forward) and 5^′^-GCTGTTGCTGGTGTAGACGG-3^′^ (reverse). Real-time PCR was performed as before.

### Western blotting

Cells were lysed in RIPA buffer with a protease inhibitor mixture and a phosphatase inhibitor mixture (Shanghai Biocolors); and lysates were run on 10% SDS-polyacrylamide gels. After transfer, the polyvinyl difluoride membranes (Millipore) were blocked for 1 h with TBS/Tween 20 containing 5% powder skim milk and then probed overnight at 4°C with primary Ab specific for cleaved Notch 1 (rabbit anti-human IgG, ABCAM). Blots were then washed five times and probed for 1 h with secondary Ab (goat anti-rabbit IgG, ABCAM). Membranes were developed with Immobilon Western Chemiluminescent HRP substrate (Millipore).

### Flow cytometry

Jurkat cells were co-cultured with DAPT for 48 hours and stained with fluorochrome-labeled mAbs against Foxp3 (eBioscience). Intracellular Foxp3 staining was performed using the Cytofix/Cytoperm intracellular staining kit, according to the manufacturer’s instructions. Flow cytometry was performed with Epics XL system (Beckman Courter) and analyzed using Expo 32 software.

### Cell viability assay

The number of viable cells was determined using a Cell Counting Kit-8 assay according to the manufacturer’s instructions (Dojindo, Japan). Cells were plated at a density of 3 × 10^4^ cells per well in a 96-well plate. After incubation for 6 hours, DAPT was added to each well at 1, 2.5, 5, 10 and 20 μM. Cells treated with 0.1% DMSO as control. After incubated for 4, 8, 12, 24, 48 and 72 hours, cells were incubated with kit reagent WST-8 for a further 2 h. The absorbance of samples (450 nm) was determined using a scanning multiwell spectrophotometer that serves as an ELISA reader.

### Cell cycle analysis

The cell cycle distribution was determined by flow cytometric analysis. Cells were re-suspended into 5 × 10^5^ cells/ml and incubated with DAPT (1, 5, 10 and 20 μM) for 48 hours. Then cells were collected and nuclear staining was performed according to the manufacturer’s instructions using Flow Cytometry Analysis of Cell Cycle Kit (GENMED, Shanghai). Following staining, cells were immediately analyzed by flow cytometry.

### Apoptosis analysis

Jurkat cells were stained with Wright-Giemsa and morphology was studied under microscopy. Apoptosis induction was confirmed using the Annexin V/PI Apoptosis Detection Kit (Jingmei Biotech, Shanghai, China). After co-cultured with DAPI, Jurkat cells were collected and washed twice with cold PBS. Cells were labeled with 5 μl Annexin V-FITC followed by10μl PI. Annexin V-PI were measured by FACS Calibur and analyzed with the Modfit Software.

### Statistical analysis

Data are expressed as mean ± SD. Statistical significance was valued by one-way ANOVA. Equal variances assumed were LSD. A *P* value less than .05 was considered statistically significant (SPSS 13.0 for windows, SPSS Inc, Chicago, IL).

## Competing interests

The authors declare that they have no competing interests.

## Authors’ contributions

XL has made substantial contributions to conception and design, and has been involved in drafting and revising the manuscript. HT have given final approval of the version to be published. YZ has participated in in vitro study and the analysis of data. TX has participated in in vivo study and the analysis of data. CW has been involved in data analysis. YL has been involved in revising the manuscript. All authors read and approved the final manuscript.
